# Predicting the response to a triptan in migraine using deep attack phenotyping: A feasibility study

**DOI:** 10.1177/0333102420959786

**Published:** 2020-09-21

**Authors:** Michele Viana, Grazia Sances, Salvatore Terrazzino, Chiara Zecca, Peter J Goadsby, Cristina Tassorelli

**Affiliations:** 1 Headache Group, Department of Basic and Clinical Neurosciences, King's College London, London, UK; 2 Neurocenter of Southern Switzerland, Neurology clinic, Headache Center, Lugano, Switzerland; 3 Headache Science Center, IRCCS Mondino Foundation, Pavia, Italy; 4 Department of Pharmaceutical Sciences, University of Piemonte Orientale, Novara, Italy; 5 Faculty of biomedical Sciences, Università della Svizzera Italiana, Lugano, Switzerland; 6 NIHR-Wellcome Trust Clinical Research Facility, King's College Hospital, London, UK; 7 Department of Brain and Behavioural Sciences, University of Pavia, Pavia, Italy

**Keywords:** Migraine, response, triptan, predictor, pain free, premonitory symptoms

## Abstract

**Background:**

Triptans, specific symptomatic medications for migraine, are not effective in a proportion of patients, or in all attacks, hence the importance of identifying predictors of response. Our aim was to investigate the association between the efficacy of oral frovatriptan 2.5 mg and clinical characteristics of migraine attacks.

**Methods:**

We enrolled 29 consecutive patients affected by migraine without aura at the Headache Center of “Mondino” Institute of Pavia. Each patient was given a diary and asked to record prospectively the features of three consecutive migraine attacks while using frovatriptan. A generalized estimating equations approach was used to determine phenotypic features associated with the pain free response at 2 hours.

**Results:**

Participants provided complete data for 85 attacks. Thirty of these (34%) patients reported being pain free 2 hours after taking frovatriptan 2.5 mg intake. Unilateral pain, presence of phonophobia, presence of one or more cranial autonomic symptoms and presence of one or more premonitory symptom were each associated with being pain free at 2 hours.

**Conclusions:**

The response to frovatriptan was associated with particular features of the migraine attack, either before or during the pain phase of attacks. The data support larger studies to explore detailed attack phenotyping, with particular attention to early signs, to enable individualized treatment in migraine.

## Introduction

Migraine has been identified as the second most disabling disorder worldwide (1). Triptans, serotonin 5-HT_1B/1D_ receptor agonists (2), provide the most effective symptomatic treatment for migraine, although both efficacy and tolerability vary among molecules within this class of drugs and between individuals, including between attacks (3–6). The basis for this variability remains to be determined.

Some attempts have been made to predict response, with frustrating outcomes (7–12). Previous studies have not included migraine premonitory symptoms, which are increasingly the subject of recognition and study (13). The potential predictive role of premonitory symptoms would be relevant since these occur in the early phase of the migraine attack, and would allow patients to treat at a point when triptans are known to be much more effective, when pain is mild (14). Moreover, some potential confounders that can modify the response to a triptan, such as previous triptan use or current use of preventive medication (5,15), were not always accounted for in previous studies.

The aim of this study was to investigate prospectively the association between different characteristics of migraine attacks and the efficacy of frovatriptan in a homogeneous population without potential confounders to test the feasibility of deeper attack phenotyping as an evolved approach to migraine treatment.

## Methods

Between October 2012 and March 2014, we enrolled consecutive patients who were seen at the Headache Science Center of “C. Mondino” National Neurological Institute of Pavia, Italy, for migraine without aura. Included patients provided their written informed consent and the study was approved by the local Ethics Committee.

### Inclusion/exclusion criteria

Participants were aged between 18 and 65 and affected for at least 1 year with headache fulfilling ICHD-2 criteria for migraine without aura (16), in use at the study initiation, and no different for our cohort than ICHD-3 (17). Participants should have had a monthly attack frequency between 1 and 5; the upper limit to avoid an immediate consideration of initiating preventive treatment.

Patients with diagnosis of medication overuse headache at any time, current diagnosis of chronic migraine, being unable to distinguish migraine from other types of headache (i.e. tension-type headache) affecting them, and patients suffering exclusively from migraine attacks with onset at wakening were excluded from participation. We excluded patients with migraine with aura to avoid any confusion with the premonitory phase. We also excluded patients with a contraindication to, or previous use of any triptan, use of opioids, use of migraine preventive medication, and history of psychiatric disorders.

### Data collection

Consecutive patients diagnosed with migraine without aura and prescribed frovatriptan 2.5 mg p.o. as acute medication were included. They were provided with an *ad hoc* headache diary and asked to record prospectively the features of three consecutive migraine attacks. Patients were instructed to use frovatriptan immediately after identifying a headache as a migraine attack regardless of pain intensity, and excluding those with an onset on wakening, and to fill their headache diary when they used their medication.

The following characteristics of migraine attacks were recorded: Location (unilateral), quality (pulsating) and intensity (on a four-point scale ranging from 3 = severe pain to 0 = absence of pain) of pain; presence of the following symptoms: Nausea and/or vomiting; photophobia, phonophobia, osmophobia, cranial allodynia; cranial autonomic symptoms (CASs), including eyelid oedema, forehead and facial sweating, conjunctival injection and/or lacrimation, nasal congestion and/or rhinorrhoea, miosis and/or ptosis; premonitory symptoms of yawning, tiredness, mood changes, neck stiffness, vertigo, nausea, photophobia, phonophobia, osmophobia, food craving, thirst, problems with concentration, speaking or reading in the previous 24 hours; and time of frovatriptan intake from pain onset.

In order to assess cranial allodynia, patients were asked if a non-painful stimulus, such as touching or washing, produced pain on their face or head.

Pain intensity 2 hours after triptan intake and the duration of the attack, marked by migraine symptom resolution, were recorded in the diary. The efficacy of frovatriptan was assessed as pain resolution within 2 hours after medication use (18). In case of pain persistence at this time point, patients were allowed to use an analgesic as rescue medication.

All patients were asked to return to the Headache Centre either when they had managed to record three consecutive attacks, or at the latest after 3 months from the initial visit.

### Data analysis

Given the aim of establishing predictability of response to a triptan within individuals, the full analysis set was defined as patients who took treatment on three attacks and provided clinical data on each attack. Variables are presented as mean ± SD, median with interquartile range, or as frequency counts (%), as appropriate. To examine the relationship between phenotypic variables of the attack and the pain-free response to frovatriptan, we used a single generalised estimating equations approach with an unstructured correlation matrix. Pain free at 2 hours was set as the binary dependent variable and fitted with a logit link function (SPSS version 26, IBM Statistics). The significance level was set at *P* < 0.05.

## Results

Thirty-nine patients were enrolled. Ten patients failed to record data for three attacks and were therefore excluded from data evaluation. The remaining 29 patients successfully recorded the features of three consecutive migraine attacks whilst using frovatriptan 2.5 mg, providing data for a total of 87 attacks. Data from two attacks out of the 87 recorded were incomplete, therefore the final dataset included 85 attacks.

### Demographics and clinical features

Of the included patients, 83% were female (25/29), mean ± standard deviation (range) age was 32.9 ± 8.3 (19–47) years. Mean age at migraine onset was 21± 11 (5–47) years, duration of illness 16.0 ± 9.5 (1–30) years. The frequency of attacks of migraine without aura per month was 3 (median, IQR: 2–4). Prior to the study, patients reported treating their migraine attacks with either paracetamol or non-steroidal anti-inflammatory drugs (NSAIDs). Twenty-seven patients exclusively suffered from migraine without aura attacks, while the remaining two patients reported infrequent episodes of tension-type headache and experienced one tension-type attack each during the observation period.

### Migraine attack features

The frequencies of the different attack features are reported in Table 1. All attacks had duration shorter than 72 hours. No patients reported symptoms of migraine aura before or during the study.

**Table 1.** Migraine characteristics of attacks (*n* = 85).

Migraine characteristics	*n* (%)
*Canonical attack symptoms*	
Unilateral pain	51 (60)
Pulsatile pain	35 (41)
Baseline pain intensity:	
• Mild	30 (35)
• Moderate	40 (47)
• Severe	15 (18)
Nausea	28 (32)
Vomiting	2 (2)
Photophobia	44 (52)
Phonophobia	35 (41)
*Other attack symptoms*	
Osmophobia	16 (18)
Cranial allodynia	18 (21)
*Cranial autonomic symptoms* (at least one)	16 (19)
Premonitory symptoms*	
PS (at least one)	54 (63)
Tiredness	32 (37)
Neck stiffness	25 (29)
Photo- / phono- / osmophobia	15 (17)
Difficulty in concentrating/reading/speaking	15 (17)
Nausea	14 (16)
Yawning	12 (14)
Vertigo/unsteadiness	9 (10)
Mood changes	7 (8)
Food craving/thirst	1 (1)
Average onset prior to headache (min)	127 ± 150 (range 10–720)

*In the last 24 hours.

### Response to medication

Patients used frovatriptan after a mean period of 63 min following head pain onset. At this time, median (IQR) pain intensity was 2 (1–3). At 2 hours, 35% (30/85) of attacks were rendered pain free.

Modeling the clinical outcome at 2 hours after frovatriptan intake: Unilateral pain (Wald χ^2^_1_ = 11.44, *P* = 0.001), presence of phonophobia (χ^2^_1_ = 4.28, *P* = 0.038), presence of one or more cranial autonomic symptoms (χ^2^_1_ = 8.42, *P* = 0.004) and presence of one or more premonitory symptom (χ^2^_4_ = 13.28, *P* = 0.010) were each associated with a pain-free response.

## Discussion

Our study suggests that deep phenotyping as a strategy to develop personalized approaches to the acute treatment of migraine is practical. Using the pain-free response at 2 hours for frovatriptan 2.5 mg as the clinical endpoint, unilateral pain, presence of phonophobia, presence of one or more cranial autonomic symptoms, and presence of one or more premonitory symptom were each associated with that outcome. Patients and physicians want better ways to associate their treatments with outcomes and are largely unsatisfied with current prescription therapies (19,20). So far, some studies have identified migraine features associated with (poor) triptan efficacy (7,8). Yet some of these features – severe pain, nausea, vomiting – are typical of the well-developed attack when triptans are less likely to be effective (14). Indeed, the collection of a more comprehensive set of migraine attack features, and the subsequent analysis with respect to frovatriptan response, suggest better prediction of outcomes is a testable question.

Migraine premonitory symptoms have hitherto not been used to predict clinical outcomes. Importantly, our data across three attacks is consistent with an analysis across single attacks treated with sumatriptan 100 mg that found unilateral pain was a predictor of a pain-free response at two hours (7).

We also found that the presence of CAS and unilateral pain are predictors of good response to a triptan. These findings are in line with two previous studies where the presence of unilateral CAS predicted good responses to sumatriptan (open study) (21) and rizatriptan (randomized, double-blind, placebo-controlled parallel-group trial) (22).

In contrast with our results, in an analysis of eletriptan and sumatriptan studies, the presence of photophobia in an attack predicted a poor response (8). Moreover, other factors have been found to be associated to response to triptans, such as the presence of nausea, vomiting, and pain severity (7,8).

Some of the varying outcomes across studies predicting treatment outcome are likely due to key design differences. First, in some studies (7,8) patients had to take their medication when pain was at least moderate in intensity, whereas in the present study patients were asked to use their medication immediately upon recognising their headache as a migraine attack. Our advice to treat the attack early may have prevented the complete phenotypic expression of the attack, reducing the frequency of occurrence of some features; that is, nausea, photophobia, or allodynia, which were accordingly identified as being lower in our patients than those reported in other studies (23,24). However, the clinical relevance of early – as compared to late – predictors of treatment response is crucial, since standard clinical advice is to treat when the patient recognises their attack. Secondly, although previous studies were conducted on larger samples of subjects, these did not minimize factors that may alter the response to treatment, such as the use of preventive medication, the previous use of triptans, or the exclusion of attacks treated at waking (5,6,18). Thirdly, previous studies included patients suffering from both migraine without or with aura (7,8,11,12), since there are reported clinical differences (25). Moreover, a recent analysis of data gathered from multiple randomised trials found that the response to sumatriptan, when used acutely in migraine attacks, was less effective in migraine with aura when compared to migraine without aura (26).

Frovatriptan has been explored in a study showing that current major depressive disorder was associated with response to medication, while generalized anxiety disorder, history of triptan intake, preventive medication and familiarity were not (12). However, despite including medical history and socio-demographical variables, this study investigated only six features of migraine attacks, and used pain relief/absence within 4 hours after the intake of frovatriptan as an endpoint, thus exposing results to a higher placebo effect rate as compared to 2 hours pain free.

The presence of both CAS and unilateral pain can represent an epiphenomenon of intense trigeminal peripheral afferent activation, which may recruit peripheral neurovascular 5-HT_1B/1D_ receptors (27). With respect to the presence of premonitory symptoms, they suggest hypothalamic involvement, which is able to facilitate the migrainous process resulting in the disinhibition of the top-down modulation of the trigeminal activity (28). Perhaps this activation through hypothalamic mechanisms is important in terms of the effect of a triptan.

### Limitations

One important limitation of our study was the small sample size. Our study aimed at screening a large set of variables on a very well characterized and unconfounded population in order to identify the relevant ones. As a feasibility study with the novelty of exploring non-canonical migraine attack symptoms, we sought to push the envelope with deeper phenotyping. Thus the number of participants was small for the breadth of phenotyping. A much larger study in terms of participants will be required to clarify these questions. Another limitation of this study design is that the complete phenotype of the attacks may not have been expressed, as frovatriptan was used early after pain onset. The frequency of some features, such as nausea or photophobia, is reported at lower rates compared to other studies, accordingly (21).

In this study, we have shown with the use of a prospective diary that unilateral pain, presence of phonophobia, presence of one or more cranial autonomic symptoms and presence of one or more premonitory symptom were each associated with the response to frovatriptan in this exploratory work. All these features are manifest in the early phase of the migraine, when the attack is more treatable by triptans. The results support exploring larger studies employing deep phenotyping to optimise acute migraine treatment.

## Clinical implications

- Unilateral pain, presence of phonophobia, presence of one or more cranial autonomic symptom and presence of one or more premonitory symptom were each associated with a response to frovatriptan.
- Early identification of factors associated with a good triptan response would potentially offer a more tailored strategy for treating acute migraine.

## References

[1] Global Burden of Disease 2016 Neurology Collaborators. Global, regional, and national burden of neurological disorders, 1990–2016: A systematic analysis for the Global Burden of Disease Study 2016. Lancet Neurol 2019; 18: 459–480. 30879893 10.1016/S1474-4422(18)30499-X PMC6459001

[2] Goadsby PJ. The pharmacology of headache. Progress Neurobiol 2000; 62: 509–525. 10.1016/s0301-0082(00)00010-1 10869781

[3] Ferrari MD Roon KI Lipton RB, et al Oral triptans (serotonin, 5-HT_1B/1D_ agonists) in acute migraine treatment: A meta-analysis of 53 trials. Lancet 2001; 358: 1668–1675. 11728541 10.1016/S0140-6736(01)06711-3

[4] Dodick DW. Triptan nonresponder studies: Implications for clinical practice. Headache 2005; 45: 156–162. 15705122 10.1111/j.1526-4610.2005.05031.x

[5] Viana M Genazzani AA Terrazzino S, et al Triptan nonresponders: Do they exist and who are they? Cephalalgia 2013; 33: 891–896. 23564209 10.1177/0333102413480756

[6] Viana M Sances G Ghiotto N, et al Variability of the characteristics of a migraine attack within patients. Cephalalgia 2016; 36: 825–830. 26498348 10.1177/0333102415613612

[7] Diener H-C Ferrari M Mansbach H; the SNAP Database Study Group . Predicting the response to sumatriptan: The Sumatriptan Naratriptan Aggregate Patient Database. Neurology 2004; 63: 520–524. 15304585 10.1212/01.wnl.0000133207.70312.30

[8] Diener HC Dodick DW Goadsby PJ, et al Identification of negative predictors of pain-free response to triptans: Analysis of the eletriptan database. Cephalalgia 2008; 28: 35–40. 10.1111/j.1468-2982.2007.01457.x 17941878

[9] Diaz-Insa S Goadsby PJ Zanchin G, et al The impact of allodynia on the efficacy of almotriptan when given early in migraine: Data from the “Act when mild” study. Int J Neurosci 2011; 121: 655–661. 21777163 10.3109/00207454.2011.605191

[10] Brandes JL Kudrow D Cady R, et al Eletriptan in the early treatment of acute migraine: Influence of pain intensity and time of dosing. Cephalalgia 2005; 25: 735–742. 16109056 10.1111/j.1468-2982.2005.00981.x

[11] Johnson HF Goadsby PJ Gelfand AA. Predictors of triptan response in pediatric migraine. Pediatr Neurol 2016; 58: 37–40. 26968976 10.1016/j.pediatrneurol.2016.01.022 PMC4899237

[10] Seo JG Park SP. Factors associated with frovatriptan response in patients with migraine: A prospective, observational study. Cephalalgia 2016; 36: 493–498. 26170008 10.1177/0333102415596443

[11] Laurell K Artto V Bendtsen L, et al Premonitory symptoms in migraine: A cross-sectional study in 2714 persons. Cephalalgia 2016; 36: 951–959. 26643378 10.1177/0333102415620251

[12] Cady RK Freitag FG Mathew NT, et al Allodynia-associated symptoms, pain intensity and time to treatment: Predicting treatment response in acute migraine intervention. Headache 2009; 49: 350–363. 19220503 10.1111/j.1526-4610.2009.01340.x

[13] Karsan N Goadsby PJ. Biological insights from the premonitory symptoms of migraine. Nat Rev Neurol 2018; 14: 699–710. 30448858 10.1038/s41582-018-0098-4

[14] Goadsby PJ Zanchin G Geraud G, et al Early versus non-early intervention in acute migraine- “Act when Mild – AwM”. A double-blind placebo-controlled trial of almotriptan. Cephalalgia 2008; 28: 383–391. 18294251 10.1111/j.1468-2982.2008.01546.x

[15] Viana M Terrazzino S Genazzani AA, et al Pharmacogenomics of episodic migraine: Time has come for a step forward. Pharmacogenomics 2014; 15: 541–549. 24624920 10.2217/pgs.14.20

[16] Headache Classification Subcommittee of the International Headache Society. The International Classification of Headache Disorders, 2nd edn. Cephalalgia 2004; 24: 9–160. 14979299 10.1111/j.1468-2982.2003.00824.x

[17] Headache Classification Committee of the International Headache Society. The International Classification of Headache Disorders, 3rd edn. Cephalalgia 2018; 38: 1–211. 10.1177/0333102417738202 29368949

[18] Tfelt-Hansen P Pascual J Ramadan N, et al Guidelines for controlled trials of drugs in migraine: Third edition. A guide for investigators. Cephalalgia 2012; 32: 6–38. 22384463 10.1177/0333102411417901

[19] Lipton RB Munjal S Buse DC, et al Unmet acute treatment needs from the 2017 Migraine in America Symptoms and Treatment study. Headache 2019; 59: 1310–1323. 31410844 10.1111/head.13588 PMC6771753

[20] Goadsby PJ. Primary headache disorders – five new things. Neurol Clin Pract 2019; 9: 233–240. 31341711 10.1212/CPJ.0000000000000654 PMC6615655

[21] Barbanti P Fabbrini G Vanacore N, et al Sumatriptan in migraine with unilateral cranial autonomic symptoms: An open study. Headache 2003; 43: 400–403. 12656712 10.1046/j.1526-4610.2003.03077.x

[22] Barbanti P Fofi L Dall’Armi V, et al Rizatriptan in migraineurs with unilateral cranial autonomic symptoms: A double-blind trial. J Headache Pain 2012; 13: 407–414. 22460943 10.1007/s10194-012-0440-y PMC3381061

[23] Poolsup N Leelasangaluk V Jittangtrong J, et al Efficacy and tolerability of frovatriptan in acute migraine treatment: Systematic review of randomized controlled trials. J Clin Pharm Ther 2005; 30: 521–532. 16336284 10.1111/j.1365-2710.2005.00677.x

[24] Savi L Omboni S Lisotto C, et al A double-blind, randomized, multicenter, Italian study of frovatriptan versus rizatriptan for the acute treatment of migraine. J Headache Pain 2011; 12: 219–226. 20686810 10.1007/s10194-010-0243-y PMC3075392

[25] Selby G Lance JW. Observations on 500 cases of migraine and allied vascular headache. J Neurol Neurosurg Psychiatry 1960; 23: 23–32. 14444681 10.1136/jnnp.23.1.23 PMC495326

[26] Hansen JM Goadsby PJ Charles A. Reduced efficacy of sumatriptan in migraine with aura vs. without aura. Neurology 2015; 84: 1880–1885. 25841032 10.1212/WNL.0000000000001535

[27] Ahn AH Basbaum AI. Tissue injury regulates serotonin 1D receptor expression: Implications for the control of migraine and inflammatory pain. J Neurosci 2006; 26: 8332–8338. 16899728 10.1523/JNEUROSCI.1989-06.2006 PMC1851888

[28] Akerman S Holland P Goadsby PJ. Diencephalic and brainstem mechanisms in migraine. Nature Rev Neurosci 2011; 12: 570–584. 21931334 10.1038/nrn3057

